# Phytochemical Profile, Extraction and Characterization of Bioactive Compounds from Industrial Hemp (*Cannabis sativa* L.) Felina 32 Variety

**DOI:** 10.3390/molecules30204148

**Published:** 2025-10-21

**Authors:** Monika Haczkiewicz, Marta Świtalska, Jacek Łyczko, Joanna Wietrzyk, Anna Gliszczyńska

**Affiliations:** 1 Department of Food Chemistry and Biocatalysis, Wrocław University of Environmental and Life Sciences, Norwida 25, 50-375 Wrocław, Poland; monika.haczkiewicz@upwr.edu.pl (M.H.); jacek.lyczko@upwr.edu.pl (J.Ł.); 2 Department of Experimental Oncology, Hirszfeld Institute of Immunology and Experimental Therapy, Polish Academy of Sciences, Weigla 12, 53-114 Wrocław, Poland; marta.switalska@hirszfeld.pl (M.Ś.); joanna.wietrzyk@hirszfeld.pl (J.W.)

**Keywords:** *Cannabis sativa* L., CBD, cannabinoids, monoterpenes, sesquiterpenes, Felina 32, hemp extracts, cytotoxic activity, phytochemical profiling

## Abstract

An efficient method for the simultaneous extraction of cannabinoids and terpenes from the leaves and flowers of *Cannabis sativa* L. (var. Felina 32) was developed. Extraction parameters, including solvent type, temperature, and pressure, were optimized, revealing that hexane enables high-yield cannabinoid recovery. Moreover, terpene composition was influenced by the extraction temperature. Two extracts with the highest cannabinoid content were selected for further study, Feli1 (64.76%) and Feli2 (61.32%), both obtained using hexane. Feli1, extracted at –55 °C, had a monoterpene-to-sesquiterpene ratio of 16.7% to 83.3%, while Feli2, extracted at 25 °C, showed a higher monoterpene content (25.2%) and lower sesquiterpene content (74.8%). Both extracts demonstrated selective antiproliferative activity against cancer cell lines, with reduced toxicity toward normal breast epithelial cells (MCF-10A). Feli2 showed slightly stronger antiproliferative effects, likely due to its higher monoterpene content. At low concentrations, both extracts stimulated the growth of MV4-11 leukemia and MDA-MB-468 triple-negative breast cancer (TNBC) cells, while higher concentrations led to growth inhibition. These stimulatory effects were weaker than those observed for pure Δ^9^-THC or CBD.

## 1. Introduction

Industrial hemp (*Cannabis sativa* L.) refers to plants in which the concentration of Δ^9^-tetrahydrocannabinol (Δ^9^-THC) in the flowers or fruiting parts does not exceed 0.3% on a dry weight basis [1,2]. The classification of *Cannabis sativa* L., initially proposed by McPartland and Small, is based on the concept of a single species comprising two subspecies: *sativa* and *indica* [1]. This classification is based on physicochemical, genetic, and morphological differences between plants. Within this system, the term “*sativa*” refers to plants with a Δ^9^-THC to cannabidiol (CBD) ratio greater than 7, typically characterized by a sweet, herbal aroma, heights exceeding 2 m, and flexible stems. In contrast, the “*indica*” designation applies to plants with a Δ^9^-THC to CBD ratio below 7, generally exhibiting a pungent odor, a sesquiterpene-dominant terpene profile, and rigid stems with heights under 2 m [1].

An alternative classification of cannabis based on chemical profiles (chemovars) was proposed by Hazekamp and colleagues, who advocated abandoning the commonly used and potentially misleading terms “*sativa*” and “*indica*”. In their studies, they demonstrated that industrial hemp types, commonly referred to as hemp, are characterized by high cannabidiol (CBD) content and low levels of Δ^9^-THC. Notably, no single terpene was identified as a specific marker for hemp varieties, whereas the examined *sativa* and *indica* strains displayed distinct and characteristic terpene profiles [3]. Another classification system based on cannabinoid profiles was proposed by Kumar et al., who described five chemotypes within the *Cannabis sativa* species. In this model, fiber-type hemp was categorized under chemotypes III and IV [4].

A variety of cannabinoid-based pharmaceutical products are already available on the market, formulated in various ratios depending on the desired therapeutic effects. Examples include Bedrocan^®^ (Bedrocan International BV, Veendam, Netherlands), Sativex^®^, (GW Pharmaceuticals Ltd., Cambridge, United Kingdom) and Marinol^®^ (AbbVie Inc., North Chicago, IL, USA) [5].

The first of these, Bedrocan^®^, is a standardized dried flower product derived from *Cannabis sativa* L., characterized by a high content of Δ^9^-THC and low levels of cannabidiol (CBD). It is recommended primarily for the treatment of neuropathic pain, spasticity associated with multiple sclerosis, and as an adjuvant therapy in certain oncological conditions. Sativex^®^ is a registered oromucosal spray containing standardized extracts of *Cannabis sativa* L. in a fixed 1:1 ratio of Δ^9^-THC to CBD. It is primarily used for spasticity associated with multiple sclerosis and for neuropathic pain resistant to conventional therapies. In contrast, Marinol^®^ is a synthetic Δ^9^-THC preparation in capsule form, used in the management of nausea and vomiting, cancer-associated anorexia and cachexia, and as part of chronic pain therapy [5]. In recent years, there has been a rapid increase in interest in low Δ^9^-THC cannabis varieties and the phytopharmaceuticals derived from them [6]. This trend is driven, on one hand, by an increasing body of scientific evidence supporting the therapeutic potential of non-psychoactive cannabinoids, such as cannabidiol (CBD) [7], and, on the other hand, by the favorable legal status of industrial hemp, which facilitates extensive experimental research and allows its use as a safe and accessible model for scientific investigation.

An additional contributing factor is the growing interest in extracts with well-defined chemical profiles, in which the synergistic interactions between cannabinoids and terpenes known as the “entourage effect” may significantly influence biological activity [8,9,10]. These preparations are considered promising candidates for the development of novel adjuvant therapies.

Felina 32 is a French variety of industrial hemp listed in the Common Catalogue of Varieties of Agricultural Plant Species [11], which includes the varieties authorized for trade within the European Union. Felina 32 is a monoecious cultivar grown for both fiber and seed production, while its inflorescences and leaves are rich in biologically active compounds, such as cannabinoids and terpenes, which have potential application in the pharmaceutical and nutraceutical industries [12,13].

In the Felina 32 variety, depending on the harvest time, the CBD concentration reaches around 1.4%, while Δ^9^-THC content does not exceed 0.2% in dry weight. Other cannabinoids, such as cannabigerol (CBG), cannabichromene (CBC), and cannabinol (CBN), are present in concentrations ranging from 0.031% to 0.310% [14].

According to Di Giacomo et al., Felina 32 may also be a source of compounds with anticancer potential, mainly due to specific terpenes in its chemical profile [15]. In essential oils extracted from Felina 32, high amounts of α-pinene, myrcene, and terpinolene were identified in the monoterpene fraction. The sesquiterpene fraction was dominated by (*E*)-β-caryophyllene, caryophyllene oxide, and α-humulene [12].

The literature indicates that extracts derived from Felina 32 have significant biological potential, largely due to their high sesquiterpene content [15]. However, previous studies mainly focused on profiling the cannabinoid fraction or on evaluating the biological activity of individual purified compounds. To date, there is a lack of comprehensive studies on developing an efficient method for the simultaneous extraction of cannabinoids and terpenes while preserving their natural chemical profiles and assessing their combined (synergistic) effects in vitro.

Therefore, the aim of this study was to develop a method for co-extracting cannabinoids and terpenes from the Felina 32 hemp variety and to obtain a preparation suitable for testing potential synergistic anticancer effects in in vitro models using selected cancer cell lines.

## 2. Results and Discussion

In recent years, an increasing number of scientific studies have confirmed the synergistic effects of cannabinoids and terpenes. Although these compounds naturally occur in cannabis, they have not been previously isolated together. Their extraction is typically performed in separate processes: solvent extraction for cannabinoids and distillation for terpenes. As a result, terpenes are often added back into cannabinoid extracts during industrial processing to enhance their commercial and biological value; however, this does not reflect the true chemical profile of the original plant variety, nor the natural proportions between these compounds.

Based on our earlier results, in which we investigated methods for the simultaneous extraction of cannabinoids and terpenes from the Futura 75 cultivar [16], we undertook the development and application of a similar approach for the Felina 32 variety. Based on the analysis of interdependencies between key process parameters, we conducted an optimization of a low-pressure solvent extraction method (LPE), enabling efficient, one-step co-extraction of both compound groups. The aim was not only to develop an effective extraction method but also, for the first time, to provide a detailed chemical characterization of the cannabinoid and terpene profile in Felina 32, preserving their natural ratios found in the plant material. In the next stage, we evaluated the biological potential of the obtained full-spectrum extracts.

### 2.1. Plant Source and Extraction Procedures

Fragmented and homogenized plant material (leaves and inflorescences) from the Felina 32 cultivar was subjected to solvent extraction to examine the effect of the extraction medium (methanol, ethanol, acetone, isopropanol, hexane, and mixtures of hexane/ethanol and hexane/isopropanol in a 7:3 *v*/*v* ratio) on extraction efficiency and the chemical profile of the isolated fractions of biologically active compounds. According to available data, both single organic solvents and binary solvent mixtures are recommended for extracting biologically active compounds from hemp [16,17].

Based on previously observed relationships between solvent polarity and pressure, the LPE process was optimized by applying higher pressure (2 bar) for polar solvents (methanol, ethanol, isopropanol), as this condition had previously resulted in increased cannabinoid extraction efficiency. In contrast, for less polar solvents (acetone, hexane, and their mixtures), lower pressure (1 bar) proved to be more effective, as it enabled longer contact between the solvent and plant material, which translated into higher cannabinoid content in the final extract.

Extractions were carried out under two temperature conditions. The first, 25 °C, was selected as a standard ambient temperature and used as a reference point. The second, –55 °C, was chosen based on the literature data and previous experimental experience, which indicate that low-temperature extraction (–40 °C to –80 °C) allows for effective isolation of both cannabinoids and terpenes while simultaneously reducing the co-extraction of undesirable compounds such as waxes and chlorophyll. This results in extracts with higher purity and improved chemical composition.

The highest extraction yield from the Felina 32 hemp cultivar was obtained using methanol as the solvent at 25 °C (0.34 g) (Table 1), which aligns with reports from the literature indicating that polar solvents, particularly, offer the highest extraction efficiency for cannabinoids [18]. The lowest yield was obtained with isopropanol at –55 °C (0.23 g). Binary solvent mixtures hexane/ethanol and hexane/isopropanol (7:3 *v*/*v*), as well as pure hexane at 25 °C, yielded comparable extract quantities, although still lower than that obtained with methanol.

In the next step, winterization of the extracts was carried out to remove wax fractions, as described in Section 3.2.2. The greatest mass loss relative to the initial extract weight was observed after extraction with hexane at 25 °C, amounting to 49% of the original extract mass. Hexane, being a non-polar solvent, promotes the extraction of waxes and lipid-like substances [19]; thus, although the loss was substantial, it is consistent with expectations [20]. Additionally, this non-polar solvent also favors the extraction of cannabinoids in their neutral form, which are more soluble in such solvents [21].

### 2.2. Profiling Cannabinoid Content in Plant Extracts

Analysis of the obtained results clearly indicates that the applied extraction parameters such as solvent type, temperature, and pressure are critical for the efficiency of cannabinoid isolation from plant material. The highest cannabinoid concentration (64.76% *w*/*w*) was achieved using hexane as the solvent at –55 °C and 1 bar pressure during extraction of the Felina 32 cultivar. The use of binary mixtures of organic solvents resulted in extracts with significantly lower cannabinoid content, ranging from 55.44% to 58.05% *w*/*w*.

Although hexane is not considered a “green” solvent, the results of our study demonstrated that it was the most effective extraction medium for the Felina 32 hemp variety, providing a high yield of cannabinoid isolation. This efficiency is attributed to its non-polar nature, which facilitates the dissolution of cannabinoids in their neutral form. In addition, the low boiling point of hexane (~69 °C) allows for its rapid removal after extraction and minimizes the risk of thermal degradation of heat-sensitive compounds, making it an attractive option from a process efficiency standpoint.

However, it is important to emphasize that hexane’s toxicological profile raises significant safety concerns. Therefore, when extracts obtained using hexane are intended for therapeutic use or as components in food products, it is essential to ensure that the final extract is completely free of solvent residues. The use of hexane thus requires additional purification steps, such as extended evaporation under reduced pressure and rigorous control of residual solvent levels in the final extract.

The concentration levels of individual extracts are presented in Table 2. The cannabinoid profile of the extracts indicates a dominant presence of cannabidiol (CBD), with a concentration exceeding 44% (*w*/*w*), while other cannabinoids such as Δ^9^-THC, CBC, and CBN were present in low amounts. The obtained cannabinoid profile is consistent with the findings reported by Ingallina et al. [14]. A similar cannabinoid profile has been observed in extracts from the Futura 75 cultivar, where CBD at 42.44% *w*/*w* also dominated over other cannabinoids [16]. Both cultivars, Felina 32 and Futura 75, are characterized by high CBD content and simultaneously low Δ^9^-THC levels (<0.3%), classifying them as chemotype III.

Based on the obtained results, it was confirmed that extracts from the Felina 32 cultivar exhibit higher total cannabinoid concentrations compared to extracts from the Futura 75 cultivar obtained under the same conditions [16].

### 2.3. Comparative Analysis of Terpene Profiles in Plant Extracts

The aim of the subsequent stage of the study was to identify terpene compounds present in the obtained extracts. This was achieved by comparing the mass spectra of the extracts with the NIST 20 (National Institute of Standards and Technology) and FFNSC 3.0 (Flavours and Fragrances of Natural and Synthetic Compounds 3.0) libraries. The calculated linear retention indices (LRi) were then compared with reference values from these databases. A total of 59 terpene compounds were identified. The complete list of these compounds is available in the Supplementary Materials (Table S1 and Figures S1–S5). To highlight the key findings, Table 3 presents the principal volatile compounds, selected based on their high abundance in the extracts.

**Figure 1:**
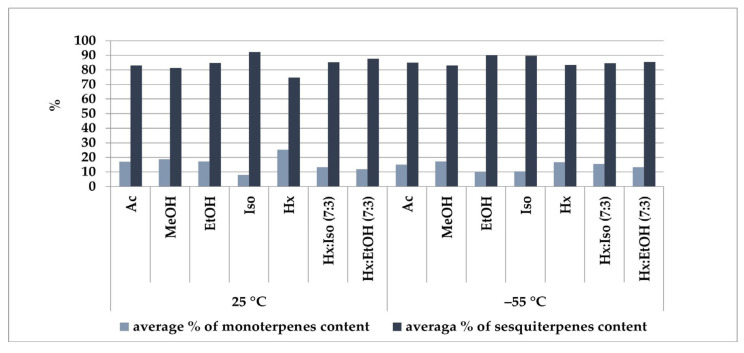
Solvent-dependent efficiency of terpene extraction from the Felina 32 hemp variety. Ac—acetone, MeOH—methanol, EtOH—ethanol, Iso—isopropanol, Hx—hexane, Hx:Iso (7:3)—hexane–isopropanol (7:3), Hx:EtOH (7:3)—hexane–ethanol (7:3), temperature (25 °C and –55 °C).

The terpene profile of the extracts was further analyzed using solid-phase microextraction (SPME) combined with GC/MS techniques, and the results are presented below in Figure 1. Analysis of the obtained chromatograms allowed for the determination of the monoterpene-to-sesquiterpene ratio in individual samples. Extracts obtained using hexane at 25 °C exhibited the highest monoterpene-to-sesquiterpene ratio, amounting to 25.21% to 74.79%. In contrast, the lowest ratio, 8.04% to 91.96%, was recorded in extracts where isopropanol at 25 °C was used as the extraction medium. Binary solvent mixtures yielded extracts in which the proportions of both terpene groups were comparable.

Fiorini and colleagues, conducting experiments on both fresh and dried plant material from the Felina 32 cultivar, confirmed that in essential oils obtained from dried material via hydrodistillation, sesquiterpenes are the dominant compounds. They also observed that drying leads to the oxidation of α-humulene and (*E*)-β-caryophyllene into humulene epoxide II and caryophyllene oxide. The monoterpene-to-sesquiterpene ratio was reported as 26.7% to 52.6%, with the total cannabinoid fraction accounting for 7.9% [12]. A predominance of sesquiterpenes was also observed in essential oils obtained via hydrodistillation by Pieracci et al., where the ratio of monoterpenes to sesquiterpenes was recorded as 2.1% to 62.8%, and the cannabinoid fraction accounted for 29.1% [22].

A comparative analysis of the terpene profiles of essential oils obtained from the Felina 32 cultivar indicates similarities in the main identified compounds: α-humulene and (*E*)-β-caryophyllene. The reported results demonstrate that hydrodistillation of dried plant material reduces the levels of sesquiterpenes, particularly α-humulene, while increasing the levels of oxidized products such as caryophyllene oxide.

In contrast to hydrodistillation, solvent extraction protects terpene compounds from degradation. In extracts obtained from the Futura 75 cultivar, significant amounts of (*E*)-β-caryophyllene (0.02–40.35%), α-humulene (4.21–33.50%), myrcene (0.33–11.50%), and α-pinene (0.01–1.16%) were observed [16]. Similarly, in extracts from the Felina 32 cultivar, sesquiterpenes (*E*)-β-caryophyllene and α-humulene were also predominant.

### 2.4. Antiproliferative Activity of Extracts Towards Cancer and Normal Cell Lines

**Figure 2:**
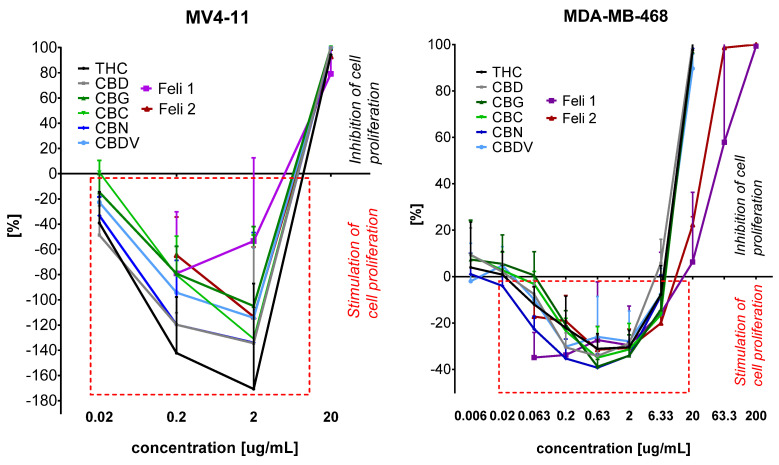
Activity of cannabinoids and Feli 1 and Feli 2 extracts against MV4-11 leukemia and breast cancer MDA-MB-468 (TNBC) cell lines.

To investigate the potential synergistic effects of cannabinoids and terpenes against cancer cell lines, two extracts with the highest cannabinoid concentrations, Feli1 (64.76%) and Feli2 (61.32%), were selected for further study. Both extracts were obtained using hexane as the extraction solvent. Feli1, extracted at –55 °C, exhibited a monoterpene-to-sesquiterpene ratio of 16.7% to 83.3%. In contrast, Feli2 extracted at 25 °C and showed the highest monoterpene content, with a ratio of 25.21% monoterpenes to 74.79% sesquiterpenes. The antiproliferative activity was investigated against six human cancer cell lines: A549 (lung) AGS (gastric), HT-29 (colon), two breast carcinomas MCF-7 (ER+), and MDA-MB-468 (TNBC) and MV4-11 (leukemia). The cytotoxicity and potential safety for their use were assessed additionally on normal human breast epithelial cells (MCF-10A). The determination of the effectiveness of these extracts in inhibiting cancer cell growth with minimal toxicity to normal cells was the aim of this study. Antiproliferative activity, after 72 h of incubation of cancer and normal cells with extracts, was assessed using the MTT assay (for MV4-11) or the SRB assay. The percentage of cell growth inhibition was calculated, and IC50 values were determined (Table 4 and Figure 2).

The Feli1 and Feli2 extracts demonstrated IC50 values in similar range (6.5–10.8 μg/mL) against a panel of cancer cell lines including lung (A549), colon (HT-29), gastric (AGS), and breast (MCF-7, ER+) cancer cells. Among them, Feli2 showed slightly higher antiproliferative activity, as indicated by its modestly lower IC50 values compared to Feli1. The lowest activity (IC50 about 30 μg/mL) was observed against breast MDA-MB-486 (TNBC) cells.

The stronger biological effect observed for the Feli 2 extract, which contains a higher proportion of monoterpenes, suggests that this fraction may be crucial for achieving greater therapeutic potential. The effect of terpenes on the increase in biological properties of cannabinoids was extensively studied by many research groups [23,24].

Analysis of the terpene profile of the Feli 2 extract indicates that the main monoterpenes potentially responsible for this activity are α-pinene and myrcene. α-Pinene is known in the literature for its anti-inflammatory and anticancer properties. In vitro studies suggest that this monoterpene inhibits cancer cell growth, mainly through the induction of apoptosis and cell cycle arrest. Myrcene, on the other hand, has been shown to possess antioxidant and cytotoxic activity against various cancer cell lines, although its exact mechanism of action has not yet been fully elucidated.

However, both Feli1 and Feli2 exhibited slightly lower antiproliferative effects against cancer cells, particularly against A549 and HT-29 cell lines, and significantly lower against MDA-MB-468 cell lines when compared to cannabinoids tested individually. Notably, despite this, both extracts displayed significantly lower cytotoxicity towards normal mammary epithelial MCF-10A cells, with IC50 values ranging from 25 to 31 μg/mL. This translated into a favorable selectivity index (SI) above 1, ranging from 2.68 to 4.51 (Table 5). Compared to the SI values of individual cannabinoids, Feli1 and Feli2 showed superior selectivity, especially towards A549 and HT-29 cancer cells. Feli1 and Feli2 and any cannabinoids tested individually showed no selectivity (SI < 1) in MDA-MB-468 cells.

When comparing the antiproliferative activity of the Felina 32 extracts to previously published results obtained from the variety of Futura 75 especially to extract Futu2, which contained 49.5% cannabinoids and a terpene composition of 19.6% monoterpenes and 80.4% sesquiterpenes, it can be hypothesized that biological activity is influenced not only by the overall cannabinoid content (which was even higher in Felina2 at 61.3%) but also by the proportion of monoterpenes to sesquiterpenes in the terpene fraction. In this regard, Feli2 contained 25.2% monoterpenes, compared to 16.7% in Feli1. Monoterpenes are widely recognized for their cytotoxic and antiproliferative properties [25]. Therefore, these findings suggest that both the specific composition of the terpene profile, including the monoterpene to sesquiterpene ratio and the total cannabinoid content, may act synergistically to influence the biological activity of hemp extracts.

The antiproliferative study of standards of cannabinoids Feli1 and Feli2 extracts showed that they, in low concentration, stimulated growth of leukemia MV4-11 and triple negative breast MDA-MB-468 cells (Figure 2).

The Feli1 and Feli2 extracts at concentrations of 0.2 and 2.0 μg/mL strongly stimulate MV4-11 cell growth (the stimulation on Figure 2 is marked as (−) values; the inhibition of growth is marked as (+) values). At a concentration of 2 μg/mL, Feli1 stimulated growth of MV4-11 cells by 60% and Feli2 by 120%, and in a lower concentration of 0.2 μg/mL, these extracts stimulated growth of leukemia cells by 70–80% (compared to untreated control cells). But the stimulation of extract was lower than stimulation, which was observed for Δ^9^-THC (170% at a concentration of 2 μg/mL and 140% at a concentration of 0.2 μg/mL) or (CBD) (140% at a concentration of 2 μg/mL and 120% at a concentration of 0.2 μg/mL). A similar observation was noticed in triple negative breast cancer cells MDA-MB-468 (Figure 2), where Feli 1 and Feli 2 at concentrations of 0.063–6.33 μg/mL stimulated cell growth in the range 20–40%, comparable to cannabinoids. The highest stimulation of growth was observed at a concentration of 0.063–2 μg/mL for Feli1 and 0.63–2 μg/mL for Feli2 (the stimulation on Figure 2 is marked as (−) values; the inhibition of growth is marked as (+) values). For concentrations higher than 6.33 μg/mL (20, 63.3, and 200 μg/mL) the inhibition of MDA-MB-468 cell proliferation was observed. And it is worth noting that these extracts and also cannabinoids stimulated growth only in the case of triple negative breast cancer MDA-MB-468 cells but no ER+ breast cancer MCF-7 cells.

It is known in the literature that cannabinoids exhibit biphasic effects on cancer cell growth, with their impact depending on the concentration. At low concentrations, Δ^9^-THC may stimulate the proliferation of certain cancer cells, such as glioblastoma cells U373-MG and lung carcinoma cells NCI-H292. This proliferative response is thought to be mediated through CB1 receptor-dependent signaling, as well as via TNF-α factor converting enzyme (TACE/ADAM17) mediated transactivation of the epidermal growth factor receptor (EGFR) [26,27].

In contrast, at higher concentrations, Δ^9^-THC and CBD show clear antiproliferative and pro-apoptotic effects. These effects are primarily mediated through activation of CB1 and CB2 receptors, induction of endoplasmic reticulum (ER) stress, and engagement of mitochondrial apoptotic pathways, including loss of mitochondrial membrane potential and caspase activation [28,29,30,31]. These biphasic effects, pro-proliferative at low concentrations and cytotoxic at higher doses, likely reflect the dose-dependent recruitment of distinct signaling cascades. At lower concentrations, cannabinoids may activate pro-survival pathways such as PI3K/AKT and ERK1/2, promoting cell growth and survival [32]. At higher doses, cellular stress responses predominate, tipping the balance towards apoptosis. Moreover, these effects may be amplified by the “entourage effect”, wherein synergistic interactions between cannabinoids and terpenes enhance the biological response. Terpenes may influence receptor binding, membrane permeability or intracellular signaling, further modulating CB1/CB2 receptor activity and the overall outcome, whether pro-survival or pro-death, depending on the extract composition, cell type, and microenvironment.

## 3. Materials and Methods

### 3.1. Chemicals and Solvents

Cannabinoid standards were obtained from Merck (Darmstadt, Germany) as 1 mg/mL methanol solutions: (–)-*trans*-Δ^9^-tetrahydrocannabinol (Δ^9^-THC), cannabidiol (CBD), cannabichromene (CBC), cannabigerol (CBG), and cannabinol (CBN). Solvents for extraction and analyses, methanol, ethanol, acetone, hexane, and isopropanol (99% *v*/*v*), were purchased from Sigma-Aldrich (Steinheim, Germany): undecan-2-one (99.9%), as well as diphenylamine (>99%), and a mixture of homologous *n*-alkanes (C7–C40). All solvents and reagents were of analytical grade.

### 3.2. Plant Source and Sample Preparation

#### 3.2.1. Plant Material

*Cannabis sativa* Felina 32 was used for the extraction, sourced from a certified agricultural farm in Poland. The combined content of (–)-*trans* Δ^9^-tetrahydrocannabinol (Δ^9^-THC) and *trans*-Δ^9^-tetrahydrocannabinolic acid (*trans*-Δ^9^-THCA) was determined to be below 0.3% (*w*/*w*). For this purpose, 30 cm apical parts of the plant were collected after the flowering stage and then dried in the absence of light at 25 °C until a moisture content of 8–12% was reached. After separating stems and seeds, the remaining plant material was ground and sieved through a 1 mm mesh. The homogenized material was then subjected to extraction.

#### 3.2.2. Low Pressure Extraction (LPE)

Pressure extraction was carried out in a low-pressure extractor equipped with a filtration system. The process was conducted at a pressure of 2 bar for methanol, ethanol, and isopropanol, and at 1 bar for acetone, hexane, and binary solvent mixtures. Nitrogen was used to force the solvent through the plant matrix. The extraction was performed at two temperatures, 25 °C and −55 °C, using methanol, ethanol, acetone, isopropanol, hexane, and solvent mixtures of hexane–ethanol and hexane–isopropanol (7:3 *v*/*v*).

The extraction parameters, including pressure and temperature for each solvent, were selected based on our previous comprehensive optimization study conducted on the *Cannabis sativa* L. Futura 75 variety [16]. In that work, it was experimentally determined that for polar solvents (methanol, ethanol, isopropanol), a pressure of 2 bar resulted in higher cannabinoid extraction efficiency. Conversely, for less polar solvents (acetone, hexane, and their mixtures), a lower pressure of 1 bar was more effective, yielding higher cannabinoid fractions [16]. The temperatures were chosen to compare extraction at a standard ambient temperature (25 °C) with a low-temperature process (–55 °C). The low temperature was selected based on the literature data, as it facilitates the isolation of cannabinoids and terpenes while minimizing the co-extraction of undesirable components such as waxes and chlorophyll, thereby increasing the purity of the final extract [16].

The extraction was performed in two stages. First, 100 mL of solvent was used to extract 10 g of plant material. The same solvent was then recirculated to extract the same plant sample a second time. Subsequently, a fresh 100 mL portion of solvent was used to rinse the plant matrix. The combined organic fractions were evaporated using a vacuum oven (Goldbrunn 450, Goldbrunn GmbH, Oberstdorf, Germany).

The resulting extracts were then subjected to a winterization process. Each extract was dissolved in ethanol (99% *v*/*v*) at a 1:10 ratio at 50 °C until clear solutions were obtained. After cooling, the solutions were stored in a freezer at −50 °C for 24 h. The extracts were filtered through filter paper placed in a chilled Büchner funnel. The wax fraction remained on the filter, while the filtrate was evaporated using a rotary evaporator under reduced pressure.

### 3.3. GC-MS Analysis of Cannabinoids in Prepared Extracts

Samples for GC-MS analyses were prepared by dissolving the final extracts in chloroform and adding internal standard (IS—diphenylamine) at a concentration of 1 mg/mL in a 1:1 ratio. The determination was performed using a previously described and validated GC-MS method [16]. Briefly, a Shimadzu QP 2020 instrument (Shimadzu, Kyoto, Japan) equipped with a column (Rxi-5ms Restek Corporation, Bellefonte, PA, USA) and parameters (30 µm × 0.25 mm × 0.25 µm) was used for the analyses. An amount of 1 µL of the mixture was injected with a split ratio (1:120) at temperature 250 °C. Helium, a carrier gas, was set with linear velocity 36.3 cm/s. The GC oven temperature was initially set at 180 °C and after 2 min of hold rose with a temperature of 35 °C/min until 320 °C with rate.

The NIST 20 database was used in the analyses. A four-point standard curve was used to compare the analytical results, prepared for each cannabinoid at concentrations of 0.01, 0.1, 0.5, and 1.0 mg/mL. The calibration curves are presented in the (Supplementary Materials).

### 3.4. Qualitative Analysis of Terpenoids in Prepared Extracts by HS-SPME

For the qualitative analysis of terpene compounds in the obtained extracts, headspace solid-phase microextraction (HS-SPME Arrow) combined with gas chromatography–mass spectrometry (GC-MS) was used following a previously described method [16]. Briefly, each extract, along with 20 μg of undecan-2-one (used as the internal standard), was placed in a vial. The prepared vials were incubated at 45 °C for 5 min to allow equilibration. The extraction phase was carried out at 45 °C for 30 min using a 1.10 mm DVB/C-WR/PDMS SPME Arrow fiber (Shimadzu, Kyoto, Japan).

Chromatographic analyses were performed using a Shimadzu GCMS QP 2020 Plus system equipped with a Zebron ZB-5 MSi capillary column (30 m × 0.25 mm × 0.25 µm; Phenomenex, Torrance, CA, USA). Helium was used as a carrier gas (linear velocity: 36.3 cm/s). The GC oven temperature program was as follows: initial temperature of 50 °C, ramped to 130 °C at 3 °C/min, then to 180 °C at 5 °C/min, and finally to 280 °C at 20 °C/min.

Compound identification was based on comparison of the obtained mass spectra with commercial spectral libraries FFNSC 3.0 and NIST20, with a minimum match threshold of 90%. The identity of each compound was further confirmed by comparing its experimental linear retention index (LRI) with the literature data, allowing a tolerance of ±15 index units. A standard *n*-alkane mixture (C7–C40) was used for LRI calibration.

### 3.5. Biological Studies

#### 3.5.1. Cell Lines and Cultured Mediums

All cell lines used were maintained at the Hirszfeld Institute of Immunology and Experimental Therapy, PAS, Wroclaw, Poland. Human cancer cell lines MV4-11 (leukemia), AGS (gastric), HT-29 (colon), and normal breast MCF-10A were obtained from the American Type Culture Collection (ATCC, USA). From the European Collection of Authenticated Cell Cultures (ECACC, UK), human cancer cell lines A549 (lung) and MCF-7 (breast) were obtained, and human MDA-MB-468 (breast) cells were obtained from the Leibniz Institute DSMZ-German Collection of Microorganisms and Cell Cultures (Germany). MV4-11 and MDA-MB-468 cells were cultured in RPMI 1640 medium (HIIET PAS, Poland) which was supplemented with 1.0 mM sodium pyruvate (only MV4-11) and 10% (MV4-11) or 20% (MDA-MB-468) fetal bovine serum (FBS) (all from Merck, Darmstadt, Germany). Cells lines A549, HT-29, and AGS were cultured in RPMI 1640 + Opti-MEM (1:1) medium (HIIET PAS, Wrocław, Poland and Gibco, Paisley, UK) supplemented with 5% FBS (Merck, Darmstadt, Germany) and 1.0 mM sodium pyruvate (only HT-29). The MCF-7 cells were cultured in Eagle medium (HIIET PAS, Wrocław, Poland) supplemented with 8 µg/mL of insulin and 1% of MEM NON-Essential amino acid (all Merck, Darmstadt, Germany). Normal breast epithelial MCF-10A cells were cultured in HAM’S F-12 medium (Corning Incorporated, Corning, NY, USA), which was supplemented with 10% Hors Serum (Gibco), 20 ng/mL EGFh, 10 µg/mL insulin, 0.5 µg/mL Hydrocortisone, and 0.05 mg/mL Cholera Toxin from Vibrio cholerae (all from Merck, Darmstadt, Germany). All culture media were supplemented with 2 mM L-glutamine (Merck, Darmstadt, Germany), 100 units/mL penicillin, (Polfa Tarchomin S.A., Warsaw, Poland), and 100 µg/mL streptomycin (Merck, Darmstadt, Germany). All cell lines were grown at 37 °C with 5% CO_2_ humidified atmosphere.

#### 3.5.2. The Antiproliferative Activity Determination

The antiproliferative activity of Feli1 and Feli2 (the solution was prepared by dissolving the substance in methanol to the concentration 50 mg/mL) were tested against six cancer cell lines. Their cytotoxicity was also tested against one normal cell line. The cells were plated in 384-well plates (Greiner Bio-One, Kremsmünster, Austria) at a density of 1 × 10^3^ (A549) or 1.5 × 10^3^ (MDA-MB-468, HT-29, MCF-7) or 2 × 10^3^ (AGS, MCF-10A) cells per well. The MV4-11 cells were plated in 96-well plates (Corning, Corning, NY, USA) at a density of 5 × 10^3^ cells per well. After 24 h, the cells were exposed in the next 72 h to Feli1 and Feli2 at the concentrations of 200.0–0.0063 μg/mL. The tested mixtures of compounds were diluted in culture medium to reach the final concentrations. The in vitro cytotoxic effect was examined using the MTT (MV4-11) or SRB assay, described previously [33]. Each compound in each concentration was tested in triplicate in a single experiment, which was repeated 3–5 times. The concentrations of Feli 1 and Feli 2, which is cytotoxic for 50% of the cells (as an IC50), were calculated for each experiment separately using Prolab-3 system based on Cheburator 0.4 software [34], and mean values ± SD are presented in Table 4 and Figure 2.

## 4. Conclusions

This study demonstrated that pressure-assisted extraction is an effective approach for the simultaneous recovery of cannabinoids and terpenes from *Cannabis sativa* L. cultivar Felina 32. The developed method enabled the isolation of extracts with a high cannabidiol content (CBD > 44% *w*/*w*) while preserving a diverse terpene profile, including major sesquiterpenes (*E*)-β-caryophyllene, caryophyllene oxide, α-humulene) and monoterpenes (α-pinene and myrcene).

Among the obtained extracts, two with the highest cannabinoid contents were selected for further cytotoxicity evaluation. The first extract (Feli1) contained 64.76% cannabinoids, with the monoterpene-to-sesquiterpene ratio of 16.7% to 83.3%. The second extract (Feli2) showed a comparable cannabinoid content (61.32%) but with a higher proportion of monoterpenes (25.2%) relative to sesquiterpenes (74.8%).

The obtained results confirm that, for *Cannabis sativa* L. cultivar Felina 32, the combination of controlled pressure, carefully selected solvent type, and process temperature adjusted to its physicochemical properties enables the maximization of lipophilic bioactive compound recovery while preserving terpene integrity. The extraction approach developed for this cultivar may serve as a basis for producing standardized, phytochemically balanced, high-quality cannabis extracts intended for potential therapeutic applications.

The biological properties of two extracts, Feli1 and Feli2, from the Felina 32-containing cannabinoids and terpene fractions with differing monoterpene-to-sesquiterpene ratios were investigated for their antiproliferative activity against cancer cells and their safety concerning normal breast cells. The results showed that the extracts had significantly higher selectivity toward cancer cells compared to non-tumorigenic cells. Furthermore, the extract with a higher monoterpene content, Feli2, demonstrated slightly higher antiproliferative activity. This fraction of terpenes appears to be crucial for the antiproliferative activity of the extract, likely through its ability to modulate oxidative stress, induce apoptosis, and enhance the cytotoxic effects of cannabinoids via synergistic interactions. But it was also observed that at lower concentrations, both extracts stimulated the growth of leukemia (MV4-11) and TNBC breast cancer (MDA-MB-468) cells, while at higher concentrations they inhibited growth of these cell lines. It is worth noting that the growth stimulation of leukemia cells by Feli1 and Feli2 was lower than growth stimulation observed for Δ^9^-THC or CBD. The therapeutic efficacy and safety are correlated with the extract composition and concentration and with type of cancer cells. These findings also provide a basis for further studies assessing the synergistic biological effects of cannabinoids and terpenes.

## Figures and Tables

**Table 1:** Mass of extracts obtained from the Felina 32 hemp cultivar before and after the winterization process (extraction conducted at a pressure of 1 bar for acetone, hexane, hexane/ethanol, and hexane/isopropanol (7:3 *v*/*v*), and at 2 bar for methanol, ethanol, and isopropanol, under two temperature conditions: 25 °C and –55 °C).

Solvent	Temp. [°C]	Mass [g] ± SD	% Mass Loss After Winterization
Ac	25	0.29 ± 0.01	–27%
	–55	0.26 ± 0.01	–17%
MeOH	25	0.34 ± 0.02	–37%
	–55	0.30 ± 0.01	–23%
EtOH	25	0.30 ± 0.02	–32%
	–55	0.27 ± 0.01	–18%
Iso	25	0.26 ± 0.01	–29%
	–55	0.23 ± 0.00	–19%
Hx	25	0.30 ± 0.01	–49%
	–55	0.25 ± 0.00	–30%
Hx:Iso (7:3)	25	0.29 ± 0.01	–29%
	–55	0.26 ± 0.00	–16%
Hx:EtOH (7:3)	25	0.31 ± 0.01	–30%
	–55	0.25 ± 0.01	–17%

Ac—acetone, MeOH—methanol, EtOH—ethanol, Iso—isopropanol, Hx—hexane, Hx:Iso (7:3)—hexane–isopropanol (7:3), Hx:EtOH (7:3)—hexane–ethanol (7:3), temperature (25 °C and –55 °C).

**Table 2:** Cannabinoid content in extracts from the Felina 32 variety obtained by pressure extraction (results given in % by weight).

Solvent	Temp. [°C]	Δ^9^-THC % Mean ± SD	CBD % Mean ± SD	CBG % Mean ± SD	CBC % Mean ± SD	CBN % Mean ± SD	% Total Cannabinoids
Ac	25	1.83 ± 0.00	49.02 ± 0.53	2.08 ± 0.08	1.98 ± 0.16	1.30 ± 0.04	56.20
	–55	1.66 ± 0.01	49.03 ± 0.08	2.11 ± 0.01	1.99 ± 0.07	1.30 ± 0.01	56.09
MeOH	25	1.66 ± 0.02	48.67 ± 0.05	2.16 ± 0.21	2.12 ± 0.26	1.29 ± 0.12	55.90
	–55	1.66 ± 0.08	48.04 ± 0.57	2.04 ± 0.05	2.42 ± 0.18	1.28 ± 0.07	55.44
EtOH	25	1.78 ± 0.16	46.86 ± 0.54	2.02 ± 0.10	1.88 ± 0.05	1.32 ± 0.02	53.86
	–55	1.86 ± 0.08	44.53 ± 0.25	1.98 ± 0.20	1.98 ± 0.20	1.28 ± 0.02	51.63
Iso	25	1.64 ± 0.06	44.87 ± 0.19	2.05 ± 0.04	1.96 ± 0.02	1.33 ± 0.02	51.85
	–55	1.73 ± 0.02	46.94 ± 0.17	2.09 ± 0.02	1.94 ± 0.19	1.25 ± 0.08	53.95
Hx	25	2.06 ± 0.09	53.98 ± 0.97	1.79 ± 0.12	2.10 ± 0.03	1.39 ± 0.02	61.32
	–55	1.84 ± 0.29	57.74 ± 0.93	1.74 ± 0.27	2.13 ± 0.65	1.31 ± 0.05	64.76
Hx:Iso 7:3	25	1.80 ± 0.03	50.67 ± 0.70	2.29 ± 0.06	2.04 ± 0.14	1.25 ± 0.08	58.05
	–55	1.64 ± 0.01	48.39 ± 0.07	2.13 ± 0.07	2.05 ± 0.16	1.23 ± 0.02	55.44
Hx:EtOH 7:3	25	1.78 ± 0.05	49.34 ± 0.074	2.22 ± 0.10	2.12 ± 0.03	1.28 ± 0.04	56.74
	–55 °C	1.87 ± 0.04	48.47 ± 0.08	2.26 ± 0.12	2.26 ± 0.12	1.22 ± 0.01	56.08

Ac—acetone, MeOH—methanol, EtOH—ethanol, Iso—isopropanol, Hx—hexane, Hx:Iso (7:3)—hexane–isopropanol (7:3), Hx:EtOH (7:3)—hexane–ethanol (7:3), temperature (25 °C and –55 °C).

**Table 3:** Main volatile organic compounds identified in the extract of Felina 32 hemp variety.

Compound	LRI_exp_^1^	LRI_lit_^2^	Content Range Min–Max [%]
α-Pinene	933	933	0.01–1.16
Myrcene	991	991	0.64–5.54
*E*-Caryophyllene	1425	1424	18.37–30.07
α-*trans*-Bergamotene	1440	1432	3.52–7.30
α-Humulene	1458	1454	28.54–31.27
Sesquisabinene	1461	1455	3.54–9.21
α-Selinene	1499	1501	2.52–3.49
Caryophyllene oxide	1589	1587	2.41–6.39

LRI_exp_^1^—experimentally calculated LRI; LRI_lit_^2^—LRI available in library.

**Table 4:** The antiproliferative activity (IC_50_ values) against selected cancer cell lines and non-tumorigenic human breast epithelial cell line (MCF-10A) after 72 h of incubation.

Compound	Cell Line/IC_50_ [μg/mL]						
	A549	MCF-7	AGS	HT-29	MDA-MB-468	MV4-11	MCF-10A
THC	3.9 ± 0.4 *	8.4 ± 1.3 *	6.4 ± 1.4 *	3.7 ± 0.6 *	11.3 ± 0.4	6.7 ± 0.2	11.1 ± 0.7 *
CBD	2.9 ± 0.2 *	3.7 ± 0.6 *	3.7 ± 0.3 *	3.0 ± 0.5 *	10.9 ± 0.8	6.33 ± 0.1	10.6 ± 0.6 *
CBG	3.7 ± 0.5 *	7.4 ± 1.4 *	6.1 ± 1.3 *	10.0 ± 1.1 *	11.6 ± 0.4	6.28 ± 0.1	10.7 ± 0.7 *
CBC	3.3 ± 0.2 *	7.6 ± 1.5 *	5.5 ± 0.9 *	5.9 ± 1.9 *	11.4 ± 0.1	6.33 ± 0.1	10.5 ± 0.5 *
CBN	3.3 ± 0.2 *	5.8 ± 1.8 *	6.2 ± 0.98 *	6.1 ± 2.1 *	11.4 ± 0.2	6.38 ± 0.1	10.8 ± 0.7 *
CBDV	6.2 ± 2.3 *	8.4 ± 2.1 *	8.4 ± 1.98 *	3.6 ± 0.4 *	12 ± 0.5	6.33 ± 0.1	11.2 ± 0.3 *
Feli 1	8.1 ± 2.6	10.2 ± 4.6	10.8 ± 1.9	8.1 ± 3.2	28.7 ± 7.7	6.8 ± 0.7	30.7 ± 4.2
Feli 2	7.2 ± 1.0	8.6 ± 0.9	9.4 ± 1.8	6.5 ± 0.8	30.5 ± 0.9	6.9 ± 0.4	25.2 ± 8.6

Data are presented as mean ± standard deviation (SD) calculated using Prolab-3 system based on Cheburator 0.4 software. * Results for free cannabinoids standards have been previously published [16].

**Table 5:** The selectivity index (SI) of tested compounds.

Compound	Cell Line/Calculated Selectivity Index SI					
	A549	MCF-7	AGS	HT-29	MDA-MB-468	MV4-11
THC	2.82 *	1.33 *	1.75 *	2.98 *	0.98	1.65
CBD	3.68 *	2.87 *	2.82 *	3.47 *	0.97	1.67
CBG	2.89 *	1.45 *	1.76 *	1.07 *	0.92	1.70
CBC	3.23 *	1.39 *	1.9 *	1.77 *	0.92	1.66
CBN	3.27 *	1.87 *	1.75 *	1.78 *	0.95	1.69
CBDV	1.81 *	1.33 *	1.34 *	3.12 *	0.93	1.77
Feli 1	3.79	3.01	2.84	3.79	1.07	4.51
Feli 2	3.5	2.93	2.68	3.88	0.83	3.65

The SI = IC_50_ for normal cell lines (MCF-10A)/IC_50_ for the respective cancerous cell line. A beneficial SI > 1.0 indicates a drug with efficacy against tumor cells greater than toxicity against normal cells. * Results for free cannabinoids standards have been previously published [16].

## Data Availability

Data are contained within the article.

## Supplementary Materials

The following supporting information can be downloaded at: https://www.mdpi.com/article/10.3390/molecules30204148/s1, Table S1. Volatile organic compounds identified in the extract of Felina 32 hemp variety; Figure S1. Calibration curve for Δ^9^-tetrahydrocannabinol (Δ^9^-THC). Linear regression equation: y = 0.3048x − 0.0036; coefficient of determination R^2^ = 0.9999; Figure S2. Calibration curve for cannabidiol (CBD). Linear regression equation: y = 1.1679x − 0.0136; coefficient of determination R^2^ = 0.9995; Figure S3. Calibration curve for cannabigerol (CBG). Linear regression equation: y = 1.1359x − 0.0114; coefficient of determination R^2^ = 0.9997; Figure S4. Calibration curve for cannabichromene (CBC). Linear regression equation: y = 0.2536x − 0.0019; coefficient of determination R^2^ = 0.9995; Figure S5. Calibration curve for cannabinol (CBN). Linear regression equation: y = 0.2581x − 0.0029; coefficient of determination R^2^ = 0.9991.
