# Supplementary material for: Phytochemical Profile, Extraction and Characterization of Bioactive Compounds from Industrial Hemp (Cannabis sativa L.) Felina 32 Variety
Source: Molecules. 2025 Oct 21;30(20):4148. doi: 10.3390/molecules30204148 (PMC12566040; doi:10.3390/molecules30204148)
Supplement: Supplementary file 1 [file molecules-30-04148-s001.zip › molecules-3861857-supplementary.pdf]

Supplementary Materials

Phytochemical profile, extraction and characterization of bioactive compounds from industrial hemp (*Cannabis sativa* L.) Felina 32 variety

Monika Haczkiewicz [1], Marta Świtalska [2], Jacek Łyczko [1], Joanna Wietrzyk [2], Anna Gliszczyńska [1,]*

[1] Department of Food Chemistry and Biocatalysis, Wrocław University of Environmental and Life Sciences, Norwida 25, 50-375 Wrocław, Poland; monika.haczkiewicz@upwr.edu.pl (M.H.); jacek.lyczko@upwr.edu.pl (J.Ł.)

[2] Department of Experimental Oncology, Hirszfeld Institute of Immunology and Experimental Therapy, Polish Academy of Sciences, Weigla 12, 53-114 Wrocław, Poland; marta.switalska@hirszfeld.pl (M.Ś.); joanna.wietrzyk@hirszfeld.pl (J.W.)

*Correspondence: anna.gliszczynska@upwr.edu.pl (A.G.)

Table S1. Volatile organic compounds identified in the extract of Felina 32 hemp variety.

**Compound**	**LRI** $_{exp}^{1}$	**LRI** $_{lit}^{2}$	**Content range min - max [%]**
α-Pinene	933	933	0.01-1.16
Benzaldehyde	960	960	0.24-2.93
Myrcene	991	991	0.64-5.54
α-Phellandrene	1005	1007	T[3]
unknown terpene	1010		0.08-1.14
α-Terpinene	1017	1018	0.04-0.16
o-Cymene	1024	1025	0.59-3.89
Sylvestrene	1028	1031	0.49-1.52
Eucalyptol	1031	1032	0.06-0.16
γ-Terpinene	1058	1058	0.39-2.16
cis-Sabinene hydrate	1067	1069	0.01-0.08
unknown terpene	1072		0.05-0.14
p-Cymenene	1090	1093	0.28-0.76
trans-Sabinene hydrate	1099	1099	0.09-0.95
Linalool	1101	1101	0.15-1.83
Fenchol	1114	1119	0.09-0.52
trans-Pinene hydrate	1122	1121	0.03-0.20
trans-p-Mentha-2,8-diene-1-ol	1136	1140	0.02-0.10
trans-Pinocarveol	1139	1141	0.09-0.59
Ipsdienol	1147	1146	0.02-0.14
unknown terpene	1158		T
Borneol	1166	1173	0.35-1.14
p-Cymen-8-ol	1186	1189	1.16-2.30
α-Terpineol	1192	1195	0.46-1.30
unknown terpene	1198		0.05-0.36
Citronellol	1229	1232	0.01-0.12
α-Ylangene	1374	1371	0.06-0.21
α-Copaene	1378	1375	0.04-0.22

β-Longipinene	1410	1407	1.07-2.15
α-*cis*-Bergamotene	1419	1416	0.05-0.95
E-Caryophyllene	1425	1424	18.37-30.07
β-*cis*-Farnesene	1435	1440	0.05-0.20
α-*trans*-Bergamotene	1440	1432	3.52-7.30
Azulene	1448	1444	0.07-0.52
Alloaromadendrene	1455	1458	0.15-0.27
α-Humulene	1458	1454	28.54-31.27
Sesquisabinene	1461	1455	3.54-9.21
9-*epi*-(*E*)- Caryophyllene	1466	1464	0.91-1.52
γ-Gurjunene	1481	1476	0.35-0.46
α-Amorphene	1484	1482	0.23-0.58
β-Chamigrene	1488	1479	0.81-1.17
β-Selinene	1490	1492	0.28-3.59
unknown sesquiterpene	1494		0.07-1.51
α-Selinene	1499	1501	2.52-3.49
β-Bisabolene	1512	1508	0.59-0.92
β-Curcumene	1515	1511	0.52-0.76
γ-Cadinene	1518	1512	0.31-0.43
7-*epi*-α-Selinene	1523	1518	0.07-0.21
β-Guaiene	1526	1523	0.19-0.26
unknown sesquiterpene	1528		0.82-1.33
Selina-4(15),7(11)-diene	1541	1540	0.35-1.07
unknown sesquiterpene	1543		T
Selina-3,7(11)-diene	1548	1546	0.47-1.21
(*E*)-Nerolidol	1567	1561	0.27-0.60
Caryophyllene oxide	1589	1587	2.41-6.39
Humulene epoxide I	1592	1605	0.71-2.03
Humulene epoxide II	1615	1613	1.25-3.65
unknown sesquiterpene	1657		0.01-1.32

LRI_{exp}[1] – experimentally calculated LRI; LRI_{lit}[2]– LRI available in library; T[3] – trace (<0.05 %)

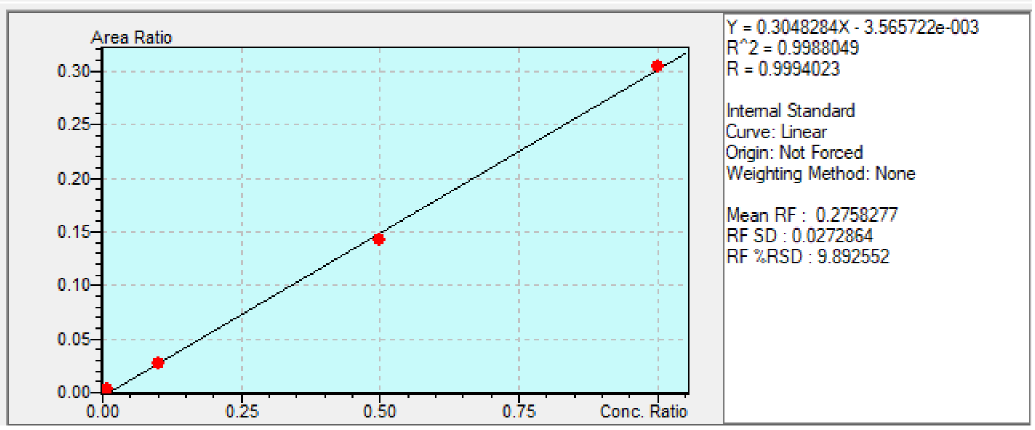

Figure S1. Calibration curve for Δ⁹-tetrahydrocannabinol (Δ⁹-THC). Linear regression equation: y = 0.3048x - 0.0036; coefficient of determination $R^2 = 0.9999$.

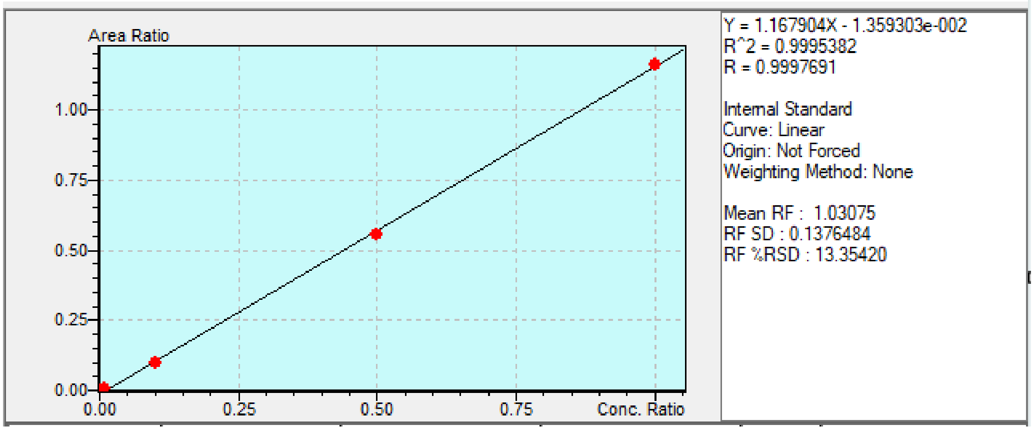

Figure S2. Calibration curve for cannabidiol (CBD). Linear regression equation: y = 1.1679x - 0.0136; coefficient of determination $R^2 = 0.9995$.

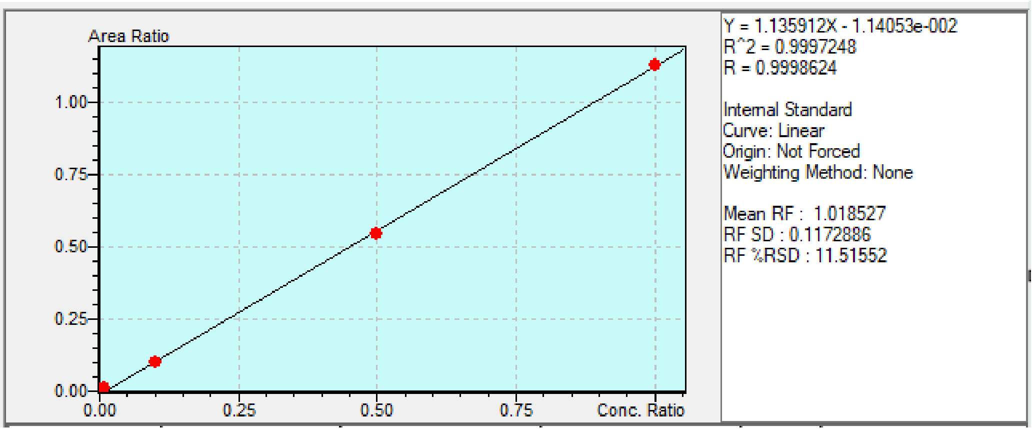

Figure S3. Calibration curve for cannabigerol (CBG). Linear regression equation: y = 1.1359x - 0.0114; coefficient of determination $R^2 = 0.9997$.

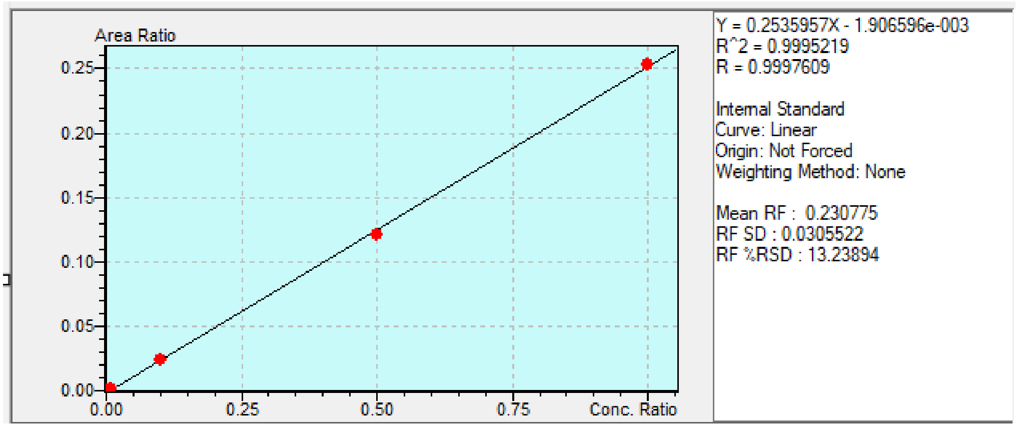

Figure S4. Calibration curve for cannabichromene (CBC). Linear regression equation: $y = 0.2536x - 0.0019$; coefficient of determination $R^2 = 0.9995$.

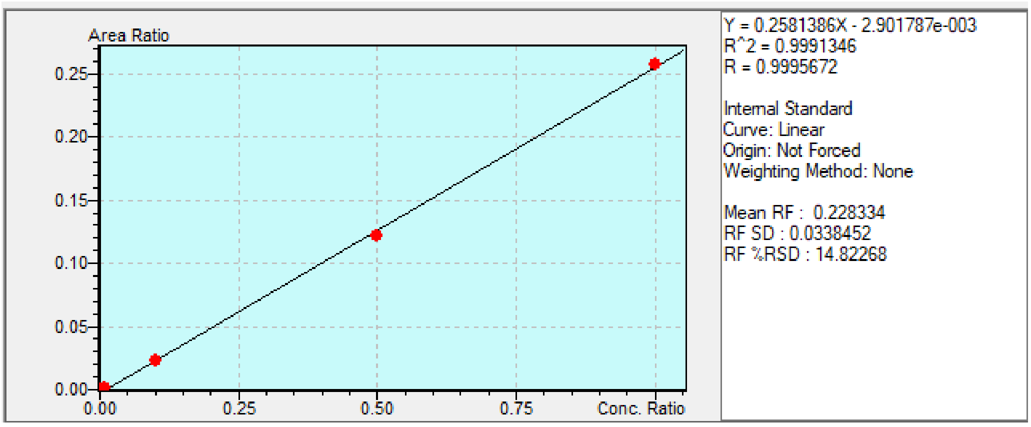

Figure S5. Calibration curve for cannabinol (CBN). Linear regression equation: $y = 0.2581x - 0.0029$; coefficient of determination $R^2 = 0.9991$.
